# Computational design of *de novo* bioenergetic membrane proteins

**DOI:** 10.1042/BST20231347

**Published:** 2024-07-03

**Authors:** Benjamin J. Hardy, Paul Curnow

**Affiliations:** School of Biochemistry, University of Bristol, Bristol, U.K.

**Keywords:** bioenergetics, protein design, transmembrane proteins

## Abstract

The major energy-producing reactions of biochemistry occur at biological membranes. Computational protein design now provides the opportunity to elucidate the underlying principles of these processes and to construct bioenergetic pathways on our own terms. Here, we review recent achievements in this endeavour of ‘synthetic bioenergetics’, with a particular focus on new enabling tools that facilitate the computational design of biocompatible *de novo* integral membrane proteins. We use recent examples to showcase some of the key computational approaches in current use and highlight that the overall philosophy of ‘surface-swapping’ — the replacement of solvent-facing residues with amino acids bearing lipid-soluble hydrophobic sidechains — is a promising avenue in membrane protein design. We conclude by highlighting outstanding design challenges and the emerging role of AI in sequence design and structure ideation.

## Why design synthetic proteins when natural ones are so fascinating?

Many (but not all) natural bioenergetic proteins comprise large multicentre or multiprotein complexes where each component is precisely arranged for catalysis and electron transport. However, the large size and sheer complexity of these assemblies can make it difficult to pick apart mechanistic details, and to derive underpinning universal principles that govern their assembly and function. Protein design offers an alternative, reductionist approach that attempts to capture at least some of the properties of these natural systems in more simple, tractable models. Such an approach has already yielded important insights into protein biophysics [1].

An additional motivation for design comes from the rapid emergence of synthetic biology and bioengineering. Synthetic biologists would like to generate minimalised cells from the top-down, construct protocells from the bottom-up, and exploit bioenergetic proteins for applications such as biosensing, biomanufacturing and bioelectronics. The size and complexity of many natural proteins means that they are not always ideal for these applications, which can also require an additional property: that such proteins be *robust* or, perhaps, *easy to work with*. This demands proteins that can be produced in common recombinant systems in large amounts, can be purified to homogeneity by straightforward methods such as affinity chromatography, are very stable and long-lived once purified, and are readily engineerable. Natural bioenergetic membrane proteins cannot always fulfil this criterion of *robustness*. The computational design of new proteins from scratch is attractive because, in principle, designer proteins can meet all the needs of the cellular engineer. They can be small and compact, have predetermined sequence composition and complexity, and be extraordinarily robust and stable. They can also be readily designed to bind redox-active cofactors for electron transport functions, and can provide good starting points for directed evolution towards a desired activity [2].

## What should we design?

**Figure 1.**
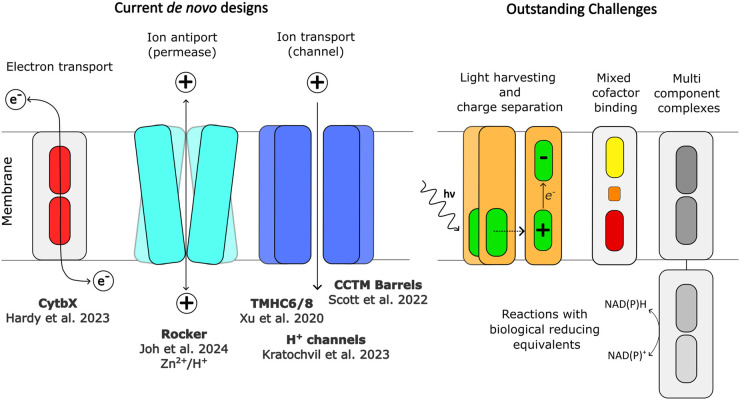
Schematic cartoons showing a broad and general depiction of current membrane protein designs, and some key design challenges that remain to be addressed.

Much of the protein design work in bioenergetics has focused on water-soluble proteins rather than their membrane-embedded cousins. New computational tools and experimental methods are now allowing membrane protein design efforts to catch up [3–5]. Although various bioenergetic design targets can be envisaged, there are generally two major goals currently being pursued. The first of these focuses on electron transport within and across lipid membrane bilayers, and this problem boils down to the positioning of redox-active centres spanning the membrane that are close enough (<25 Å) to allow spontaneous electron transport. The second target is the directional transport of protons and other ions from one side of the membrane bilayer to the other, in order to generate or dissipate electrochemical gradients (Figure 1).

Bilayer-spanning integral membrane proteins come in two flavours. The first of these is the α-helical bundles. For these proteins, the membrane-embedded polypeptide is biased towards amino acids with lipid-soluble, hydrophobic sidechains. An α-helical structure is formed spontaneously within the membrane in order to satisfy main-chain hydrogen bonds. In principle, almost any hydrophobic peptide sequence of sufficient length — ∼20–30 amino acids — will form an α-helix across a bilayer. More specifically, bundles composed of four transmembrane helices appear to represent a minimal architecture that can support a variety of functions (discussed below). Alternatively, β-barrel proteins are found in the outer membranes of bacteria, mitochondria and chloroplasts. Barrels form to satisfy hydrogen bonding between antiparallel transmembrane β-strands, and their sequences can feature a higher proportion of polar residues that face the barrel lumen. While transmembrane β-barrels have been designed [6], most attention has been paid to the α-helical proteins since they are more common in nature, have a greater variety of functions, and the α-helix is generally more designable (i.e. we have a clear understanding of the principles that govern the folding and assembly of the α-helix, and the same helical tertiary fold can be defined by multiple different sequences).

Many computational designs have been realised through the use of chemically-synthesised peptides, which can spontaneously self-assemble within model lipid bilayers or surfactant micelles. However, the advent of affordable commercial gene synthesis now offers the opportunity to genetically encode designer membrane proteins for recombinant production in live cells. This can probe aspects of the biosynthetic machinery involved in membrane protein folding and assembly, allow designed proteins to engage with cellular biochemistry, facilitate directed evolution and library screening, and enable the study of larger single-chain polypeptides.

## Artificial cytochromes for electron transport

Integral membrane cytochromes bind the redox cofactor heme and are fundamental to respiratory and photosynthetic electron transport. In membrane cytochromes, non-covalent (*b*-type) heme is commonly bound in the six-co-ordinate state via bis-His ligation to the central iron atom. Most transmembrane cytochromes bind to two hemes that are within 15 Å of each other in the membrane domain, to allow for rapid electron transport between the cofactors and across the bilayer. One of the most important characteristics is the heme redox potential, which describes the propensity of the heme prosthetic group to accept or donate electrons. This property is closely linked to the reactivity of the cytochrome and its role within an electron transport chain. The heme redox potential can be modulated by several factors including the cofactor orientation, the immediate protein environment and coordination of the central iron [7].

There has been considerable success in the rational design of transmembrane cytochromes. This has largely come from the engineering of membrane peptides and integral membrane proteins for heme binding [8,9] or from the rational conversion of soluble heme-binding peptides into membrane-embedded constructs [10–12]. An early study by Cordova et al. [13] used computational modelling and ligand docking to convert the well-studied transmembrane domain of glycophorin A into a homodimeric heme-binding peptide known as ME1.^1^
^1^There is no systematic nomenclature for designed proteins. The given name for any design is largely at the discretion of the designer. A model was built for a 32-residue hydrophobic peptide dimer based upon an existing NMR structure for the wild-type sequence of glycophorin A. (The model was built and analysed using the programme InsightII; modern alternatives for graphical model-building might be Discovery Studio, ChimeraX or PyMOL). Heme was manually docked into a hydrophobic cleft at the interface between the two helices, with the propionate groups facing the membrane exterior, and two residues within the protein interior were changed to Histidine to enable bis-His coordination of the central heme iron. Other sequence changes included residue substitutions to minimise observable steric clashes between the protein and the heme, and to enable electrostatic interactions between the peptide and the heme propionates. Finally, the model was energy-minimised with the ESFF forcefield [14]. (Generally, a high-quality energy-minimised model — preferably based on an experimental structure — is a required starting point for design).

A synthetic peptide corresponding to the ME1 sequence was found to self-assemble in surfactant micelles and to successfully complex a single heme group, with a redox potential of −128 mV at pH 7.2. The heme-protein complex was found to be catalytically active as a heme peroxidase. Since the peptide was designed for complete coordination of the central heme iron, this activity suggests a degree of conformational fluctuation in the assembled complex that allows access to a catalytically-active 5-coordinate state [15]. We have observed similar behaviour in our own work [9,16].

Other design studies have now demonstrated the effectiveness of computational surface-swapping; where charged and polar amino acids at the surface of water-soluble folds are substituted with hydrophobic residues that are compatible with the membrane interior. This approach is underpinned by the preservation of intraprotein contacts that define the protein fold and any cofactor binding site(s) between the water-soluble and membrane-integral versions of the protein. On the face of it this strategy would appear to be rather simplistic, but in fact has now been used to produce several different *de novo* membrane proteins [17–20].

One example of this comes from the design of PRIME, a self-assembling peptide that was intended to co-ordinate two non-natural porphyrins within a homotetrameric transmembrane bundle. PRIME was based upon a *de novo* water-soluble bundle that binds two molecules of Fe(III) diphenylporphyrin. Helix packing interactions in this bundle were mediated by an ‘Ala-coil’ motif, which can be found in both water-soluble and membrane proteins. A computational model was first built according to idealised backbone parameters describing a D2-symmetrical 4-helix bundle. A His residue was introduced for direct ligation to the porphyrin iron, with a specific Thr residue stabilising the axial His conformer via hydrogen bonding. After computational sequence design of the rest of the peptide, designs were scored with the CHARMM22 forcefield using an implicit membrane model (IMM1). The resulting 24-residue PRIME peptide cooperatively self-assembles around the porphyrin cofactors in surfactant micelles and in model lipid bilayers, and the two diphenylporphyrins have innate redox cooperativity with split redox potentials measured at −97 ± 3 and −168 ± 3 mV.

**Figure 2.**
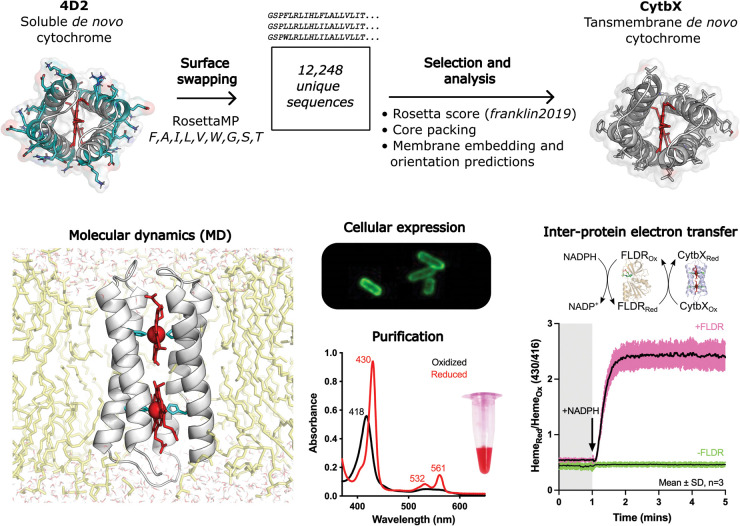
The *de novo* integral membrane cytochrome CytbX, designed via surface-swapping of a water-soluble bundle. A snapshot is shown from a MD simulation of the diheme holoprotein in a lipid bilayer. This construct is produced recombinantly in *E. coli* and purified in the diheme form. The heme cofactor takes part in protein-protein electron transfer with the promiscuous flavoprotein FLDR. All images adapted from ref. [21].

Our own recent work has shown that *de novo* cytochromes are compatible with biological systems [21] (Figure 2). We applied computational surface-swapping to the *de novo* protein 4D2, a small water-soluble 4-helix bundle that binds two molecules of *b*-type heme and has an experimental crystal structure at 1.9 Å resolution [22]. This study utilised the membrane framework of the Rosetta software, known as RosettaMP [23,24], with an updated energy function (*franklin2019*) that gives a more realistic representation of the membrane environment by using an implicit solvent model for the membrane that incorporates experimental data from protein folding studies [25,26]. All protein-heme interactions were retained in the bundle core, and critical intraprotein interactions were maintained by preserving residues that were engaged in knobs-into-holes packing at helical interfaces. Residues at the exterior of 4D2 were allowed to sample a reduced amino acid alphabet (FAILVWGST) to match the hydrophobic medium of the membrane. Interhelical loops were adjusted to conform to the ‘positive inside rule’, whereby regions of membrane proteins that face the cell interior carry an overall bias for the positively-charged sidechains of Lys and Arg [27]. The heme orientation was maintained so that propionate groups were oriented towards the membrane exterior and could interact with positively-charged sidechains as for ME1. We produced 12 248 unique design sequences using a flexible backbone protocol in RosettaMP over three days of computational time across 20 CPU nodes of a high-performance computing cluster. We selected three designs for further study based on packing statistics and their overall Rosetta score. Molecular dynamics (MD) simulations in *Escherichia coli* lipids were performed for each design using Amber18 [28] to interrogate protein dynamics and fold stability with and without heme; this is a useful screening step since, generally, well-packed designs exhibit low conformational dynamics in MD. (For an overview and discussion of membrane MD see [29]). Each of these three designs was encoded as a synthetic gene and all were successfully expressed in *E. coli*. One of the designs was produced at relatively high levels in the cell, and became the focus for further characterisation; we termed this high-expressing design CytbX. We demonstrated that CytbX integrates into cell membranes and becomes loaded with endogenous heme *in situ*. CytbX can be readily purified in solubilising detergents and exhibits the extreme robustness that is a common property of *de novo* proteins. All properties of CytbX are consistent with the intended design and the two hemes of CytbX show exciton coupling, with experimental split redox potentials of −14.1 ± 0.6 and −127 ± 0.6 mV similar to values observed in natural diheme cytochromes [30]. Purified CytbX engages in protein-protein electron transfer with the *E. coli* flavodoxin reductase and has nascent quinone reactivity, making this construct a good starting point for directed evolution or further design towards a synthetic membrane-bound quinone oxidoreductase. Overall, CytbX could be a robust transmembrane electron-transport hub for building synthetic bioenergetic systems.

## Ion transport: permease-like

One critical function of bioenergetic proteins is the ‘generation of difference’ — establishing and maintaining non-equilibrium electrochemical gradients across membranes. Proton gradients are of particular interest since they can power transporter proteins for the uptake of small molecule nutrients, as well as drive the synthesis of ATP via the proton transport-coupled ATP synthase.

The DeGrado lab produced a transmembrane 4-helix bundle — ‘Rocker’ — that could act as a Zn^2+^/H^+^ antiporter [31,32]. This represents one of few *de novo* proteins with explicitly designed dynamics that can underpin function; namely, the oscillation between different structures in the membrane to either bind or release substrate. Rocker comprises a 25-residue synthetic peptide that can self-assemble into a membrane-embedded homotetramer. Design began with a computational search for backbone configurations that could form a symmetrical four-helix bundle. This backbone model was then decorated with residues that would produce two 4Glu2His di-metal binding sites at either end of the bundle. All other positions were packed by iterative rounds of sequence design biased towards hydrophobic amino acids. A key aspect of this process was the deliberate introduction of structural asymmetry by flaring the assembled bundle at either end, allowing conformational transitions around a central fulcrum that can transfer metals from one binding site to the other. The resulting designs were scored with CHARMM-31 using the IMM1 forcefield and an energy function which favoured bilayer-compatible designs that retained the ‘flaring’ asymmetry. MD simulations were used to assess the conformational mobility of the assembled bundle and the potential transport function.

Rocker was produced as a synthetic peptide and found to assemble into the designed antiparallel homotetramer in both micelles and lipid bilayers. X-ray crystallography confirmed the structure of the non-metallated state and Zn^2+^ binding was observed by NMR. Transport assays were performed after reconstituting Rocker in synthetic liposomes. Rocker was able to transport Zn^2+^ and Co^2+^ across proteoliposome membranes down a concentration gradient and was also found to simultaneously transport protons in the opposite direction (Zn^2+^/H^+^ antiport). Accordingly, a proton gradient could also provide the energetic driving force for Zn^2+^ transport, although the precise transport mechanism remains to be confirmed [32].

## Ion transport: channel-like

**Figure 3.**
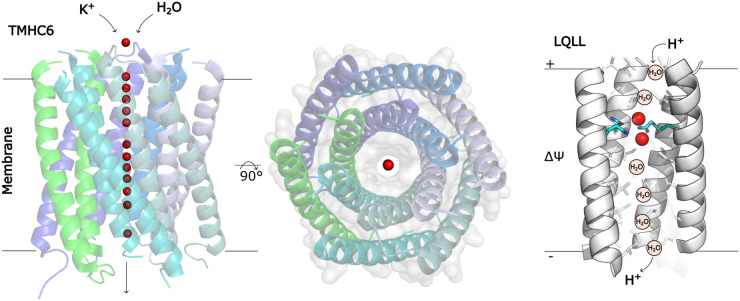
Depictions of two *de novo* designed transmembrane channels. *Left*: The TMHC6 channel [18], with helical hairpins coloured by chain, and crystallographic water molecules spanning the pore shown in red. *Right*: The ‘LQLL’ proton-transporting channel [36], with proton-loading site (PLS) residues shown in cyan sticks, hydrophobic pore residues shown in grey sticks, and ordered crystallographic waters shown in red. Protons are transported through the channel via transient ordered water wires centred around the PLS. The structure is a pentamer; the fifth helix is hidden for display.

There have been several reports of transmembrane ion channels formed from sequence-minimal *de novo* peptides [33–35]. A recent study [36] developed transmembrane proton channels from an existing pentameric *de novo* membrane protein with a narrow pore [37]. One of these channel designs, LQLL, is shown in Figure 3. The channel sequences were designed on a rational basis by replacing some of the hydrophobic pore-lining residues with clusters of glutamines that could act as polar proton loading sites. MD simulations were used to evaluate the likely functionality of the designs, X-ray crystallography confirmed the expected structure, and proton transport was observed in reconstituted proteoliposomes. Proton transport down the channel is expected to occur via the ‘proton hopping’ or Grothuss mechanism observed in natural bioenergetic proteins such as cytochrome c oxidase and ATP synthase [38], whereby protons travel along a relatively static chain of ordered waters. Transport by this mechanism allows proton transport without the collapse of other bioenergetic gradients. Overall, the design principles implemented in this study provide a very promising route towards bioenergetic proton channels.

Xu et al. [18] reported the computational design of two transmembrane channels by surface-swapping of soluble α-helical barrels. They identified single polypeptides with a hairpin fold that could self-assemble into large ring-like multimers such that one helix of each polypeptide hairpin contributed to an inner ring facing the channel lumen, and the other helix of the hairpin formed an outer ring exposed to solvent. The interfaces between the inner and outer rings were dictated by extensive hydrogen bonding networks introduced using Rosetta HBNet, and computational sequence design was performed in Rosetta for a smaller 12-helix channel and a larger 16-helix channel. Each of these channel designs was experimentally validated in their water-soluble form before hydrophobic surface-swapping was used to transform them into integral membrane proteins. These were genetically-encoded and produced in recombinant bacterial and insect cells. The small channel design (TMHC6; Figure 3) was found to be selective for the transmembrane passage of K^+^ over other ions, while the larger channel allowed the passage of small molecules such as fluorescent dyes. A hybrid rational-computational design approach was also used by the Woolfson group to produce transmembrane channels. This also employed a surface-swapping approach, where the surfaces of water-soluble hexameric peptide barrels were substituted with Leu and Trp for compatibility with the membrane core and headgroup regions respectively, and the peptide length was extended to match the dimensions of a lipid bilayer [19]. Peptides corresponding to these designs could self-assemble to form selective cation channels in model bilayer systems.

## Future directions and outstanding challenges

The studies reviewed here emphasise that surface-swapping is an effective approach in membrane protein design. Such an approach has enabled the successful design of functional membrane proteins that transport electrons, protons, ions and small molecules. It will be of interest to extend this to more sophisticated functions such as charge separation and light harvesting. This is likely to be facilitated by the binding of other cofactors such as chlorophylls, substituted porphyrins and chlorins, carotenoids, bilins, flavins and metal clusters [39–42] (Figure 1). Of particular interest is the recent demonstration that a water-soluble coiled-coil which binds a trio of cofactors — a photoexcitable pigment, an electron acceptor, and an electron donor — can act as a minimal photochemical reaction centre [40]. However, key challenges remain in the assembly of proteins around multiple cofactors [10,43] and in the incorporation of more exotic cofactors when proteins are expressed in common recombinant cell lines. Building electron transport chains will require careful control over the distance between redox centres and redox tuning will be essential to ensure rapid, productive, directional electron transfer. For example, to construct multiheme cytochromes capable of long-range extracellular electron transport like those found in *Shewanella oneidensis* and *Geobacter sulfurreducens*, one could combine designer transmembrane cytochromes such as CytbX with pre-designed water-soluble multiheme wires such as the tetraheme e4D2 [22]. Efficient electron conductance would be reliant on the order of heme potentials, and the design would need to straddle the outer cell membrane. Interfacing designer proteins with biological reducing equivalents such as nicotinamides will likely necessitate the design of flavin cofactor binding sites, an active area of research. Additional functionality such as quinone cycling could be introduced to *de novo* cytochromes by binding-site design or directed evolution.

A further outstanding challenge remains to routinely design membrane proteins that can undergo specific conformational transitions [44], especially because design methods tend to favour the stability of a single conformation. This issue may be less pronounced for some cofactor-binding proteins but will be critical for multi-state design to mimic some multicentre electron transport proteins, gated channels and solute transporters. A pertinent example from a non-bioenergetic system is the functional redesign of natural membrane receptors by targeting specific residues involved in allosteric coupling [45]. Additionally, it is not clear how easily the surface-swapping approach can be applied to more complex topologies and folds that include, for example, highly tilted helices and re-entrant loops. A combination of computational tools with laboratory screening methods and directed evolution may be required to more fully explore the structural and functional fitness landscape.

Whilst the membrane protein capabilities of classical computational tools such as Rosetta are still under active development [46] it seems likely that a major impact will be seen from the emergence of generative AI and language models for protein design [47–49]. Tools including LigandMPNN [50], RoseTTAFold All-Atom [42], Chroma [51] and RFDiffusion [42,52], have immense potential for rapidly generating novel designs that incorporate stipulated cofactors. These tools are not yet fully tested with membrane proteins. Importantly, generative AI models will allow designers to move away from simple four-helix bundles to more diverse folds that may be more suitable for scaffolding multiple or more complex cofactors. In parallel to these design tools, new structure prediction methods that specifically incorporate small molecule ligands will enable the more accurate assessment of ligand-binding designs. The continual development of accessible gene synthesis should further encourage the design of recombinant *de novo* membrane proteins that coexpress and co-operate with natural proteins for cell engineering. These exciting developments mean that the stage is now set for rapid progress in the design of non-natural membrane proteins with true bioenergetic capability.

## Perspectives

- The design of bioenergetic membrane proteins is of keen interest as a means to unpick the fundamental principles of membrane protein assembly and function; and as a route towards robust modular components for applications in synthetic biology and bioengineering.
- Current thinking uses rational design or classical computational tools (e.g. Rosetta) and a major focus has been on the resurfacing of soluble protein folds to produce membrane-compatible helical bundles. These design efforts have been successful in producing cytochromes, ion channels and metal transporters.
- Future directions will include de novo folds and novel sequences that can co-ordinate a variety of bioenergetic pigments and larger constructs (or assemblies) that can house multiple functional centres. This will probably be enabled by a series of machine learning tools that are dedicated to protein design and can incorporate small molecule ligands.

## Open Access

Open access for this article was enabled by the participation of University of Bristol in an all-inclusive *Read & Publish* agreement with Portland Press and the Biochemical Society under a transformative agreement with JISC.

## Abbreviations

MD: molecular dynamics
PLS: proton loading sites
